# Vitamin K antagonists and cardiovascular calcification: A systematic review and meta-analysis

**DOI:** 10.3389/fcvm.2022.938567

**Published:** 2022-08-19

**Authors:** Nina D. Kosciuszek, Daniel Kalta, Mohnish Singh, Olga V. Savinova

**Affiliations:** ^1^New York Institute of Technology, College of Osteopathic Medicine, Academic Medicine Scholar Program, Old Westbury, NY, United States; ^2^Department of Biomedical Sciences, New York Institute of Technology College of Osteopathic Medicine, Old Westbury, NY, United States

**Keywords:** cardiovascular calcifications, atherosclerosis, coronary artery disease, breast arterial calcifications (BAC), peripheral arterial disease (PAD), aortic calcification index, carotid atheroma, calcific aortic valve disease (CAVD)

## Abstract

**Background:**

Many patients treated with Vitamin K antagonists (VKA) for anticoagulation have concomitant vascular or valvular calcification. This meta-analysis aimed to evaluate a hypothesis that vascular and valvular calcification is a side-effect of VKA treatment.

**Methods:**

We conducted a systematic literature search to identify studies that reported vascular or valvular calcification in patients treated with VKA. The associations between VKA use and calcification were analyzed with random-effects inverse variance models and reported as odds ratios (OR) and 95% confidence intervals (95% CI). In addition, univariate meta-regression analyses were utilized to identify any effect moderators.

**Results:**

Thirty-five studies were included (45,757 patients; 6,251 VKA users). The median follow-up was 2.3 years [interquartile range (IQR) of 1.2–4.0]; age 66.2 ± 3.6 years (mean ± SD); the majority of participants were males [77% (IQR: 72–95%)]. VKA use was associated with an increased OR for coronary artery calcification [1.21 (1.08, 1.36), *p* = 0.001], moderated by the duration of treatment [meta-regression coefficient B of 0.08 (0.03, 0.13), *p* = 0.0005]. Extra-coronary calcification affecting the aorta, carotid artery, breast artery, and arteries of lower extremities, was also increased in VKA treated patients [1.86 (1.43, 2.42), *p* < 0.00001] and moderated by the author-reported statistical adjustments of the effect estimates [B: −0.63 (−1.19, −0.08), *p* = 0.016]. The effect of VKA on the aortic valve calcification was significant [3.07 (1.90, 4.96), *p* < 0.00001]; however, these studies suffered from a high risk of publication bias.

**Conclusion:**

Vascular and valvular calcification are potential side effects of VKA. The clinical significance of these side effects on cardiovascular outcomes deserves further investigation.

## Introduction

It is well-recognized that vascular calcification is an independent predictor of cardiovascular disease (CVD) and mortality (1). Studies have shown that calcification correlates with clinically-significant coronary artery disease (CAD) (2–4), acute cardiac and cerebrovascular events (5, 6), arterial stiffness and hypertension (7), and aortic valve disease (8). CVD is the leading cause of death, accounting for over 30% of mortality worldwide. Coronary artery calcium scoring has emerged as a non-invasive imaging platform for atherosclerotic CVD risk stratification and guiding lipid-lowering therapies for primary prevention (9, 10).

Warfarin, a vitamin K antagonist (VKA), was introduced into clinical practice as an anticoagulant in the 1950 s (11). Over the years, warfarin and other VKAs have been approved for the prophylaxis of thrombotic events in recurrent venous thrombosis, atrial fibrillation, valvular heart disease, and valve replacement (12). Although the use of VKA has declined in the past few years due to the introduction of safer non-vitamin K oral anticoagulants [NOAC or DOAC (direct oral anticoagulants)], VKAs remain widely prescribed and is the only guideline-recommended therapy for patients with prosthetic valves (13–16). Moreover, older patients and patients with comorbidities are more likely to receive warfarin for anticoagulation (17).

VKA inhibits coagulation factors II, VII, IX, X, and several other proteins by suppressing vitamin K-dependent post-translational gamma-carboxylation required for their function (18). Calcification is suppressed under normal physiologic conditions by several endogenous inhibitors, including matrix Gla protein (MGP), pyrophosphate, and plasma fetuin-A (19). MGP belongs to the same group of gamma-carboxylated proteins as coagulation factors and requires gamma-carboxylation for its inhibitory activity (18). Long-term use of VKA is associated with increased vascular calcification, presumably due to the reduction of vitamin K-dependent gamma-carboxylation of MGP (20, 21).

The role of VKA in vascular calcification is still poorly recognized, and the clinical significance is undefined (22). Here, we present the first meta-analysis of clinical studies on this topic. We aimed to provide objective evidence for the association between VKA use and cardiovascular calcification.

## Methods

### Search strategy

All clinical studies except case studies and case series were considered, and the inclusion was not limited to a specific indication for VKA use. Primary outcomes were coronary artery calcification, extra-coronary calcification (abdominal or thoracic aorta, carotid arteries, breast arteries, and arteries of the extremities), and valvular calcification. The core systematic literature search was conducted in PubMed with a stepwise keyword search strategy (Supplementary Table 1) up until March 29, 2022. The search results were filtered using pubmed filters to exclude reviews, case reports, guidelines, and study protocols. The reference lists of the relevant articles and “similar articles” suggested by pubmed were also considered. Other databases, including CINAHL, cochrane register of studies, and google scholar, were searched for additional references. Abstracts were screened for the inclusion criteria: (1) VKA treatment and (2) at least one vascular or valvular calcification outcome, e.g., calcium score or index, calcified plaque volume, presence/absence of calcification, calcification severity grade, or an annual rate of progression. Two investigators screened the abstracts, and any disagreements were resolved by finding a consensus.

### Data extraction and management

Data were selected based on a full-text assessment. The extracted data included a study identifier, country, study design, sample size, mean or median age, percent of males, VKA treatment or exposure, duration of VKA therapy, calcification outcome(s), methods of assessment of calcification, effect size estimates, and a brief description of statistical models used for the effect estimates. The effect sizes were extracted as incidence, prevalence, odds ratios (OR), mean change from baseline, regression coefficients, ratios of expected counts (REC), and F statistics. The coronary outcomes were coronary artery calcium (CAC) score, measured *via* computed tomography (CT), calcified plaque volume determined by coronary CT angiography (CCTA), and CAC index obtained *via* intravascular ultrasound (IVUS). Extra-coronary outcomes were the presence or absence of calcification, severity grade, calcification score, or an annual rate of progression (detected by CT, X-ray, mammography, or histopathology). Lastly, the aortic valve calcification outcomes included the presence or absence of calcification on transthoracic ultrasound (US), the number of affected aortic valve leaflets, CT calcification score, or positive findings on histopathology.

### Risk of bias assessment

The risks of bias were assessed using the Revised Cochrane risk of bias tool (RoB 2) for randomized trials (downloaded February 9, 2022, from https://sites.google.com/site/riskofbiastool/welcome/rob-2-0-tool/current-version-of-rob-2) (23) or the Newcastle—Ottawa quality scale (NOS) (24) for observations studies.

### Statistical analysis

Data were analyzed using the Review Manager computer program, Version 5.4 (RevMan5, the Cochrane Collaboration, 2020). Effect sizes were expressed as OR and standard errors (SE) using the RevMan5 effect size calculator or an online effect size calculator tool [Practical Meta-Analysis Effect Size Calculator (25)]. Inverse-variance random-effects models were used for data synthesis. Studies were grouped according to the site of calcification, coronary, extra-coronary, and valvular. The combined estimates were calculated as OR and 95% confidence intervals (95% CI) for the presence of vascular or valvular calcification in VKA-treated patients compared with other patients (non-VKA), which included patients treated with non-VKA anticoagulants and those who had no indications for anticoagulation and were not treated with any anticoagulants. The statistical heterogeneity was evaluated using the *I*^2^ test calculated in RevMan5. The risk for publication bias was assessed by an Egger regression and Begg & Mazumdar rank correlation tests, using the Meta-Essentials tool (downloaded on February 9, 2022, from https://www.erim.eur.nl/research-support/meta-essentials) (26). The Meta-Essentials tool was also used for the univariate meta-regression analyses considering the year of publication, geographic region (continent), study design, sample size, patient characteristics, median age, a ratio of participants by sex, duration of VKA treatment, calcium imaging modality, and whether or not the effect estimates were adjusted for confounders. Sub-group analyses were conducted according to each significant modifier detected by meta-regression. Furthermore, the sensitivity analysis was performed by excluding one study at a time from the corresponding meta-analysis. The significance was accepted at *p* < 0.05.

## Results

### Search results and characteristics of the included studies

**Figure 1:**
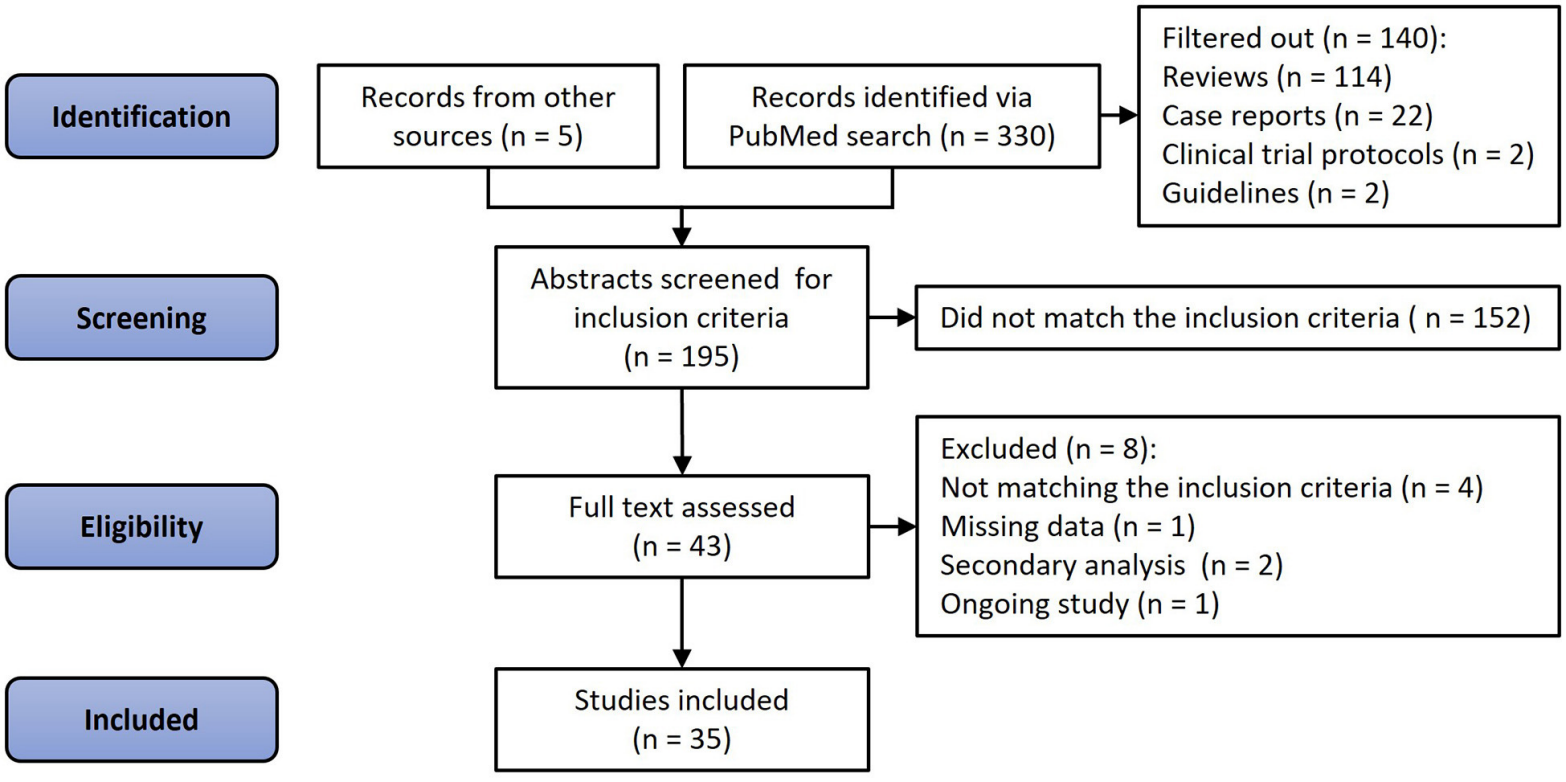
Flow diagram of the literature selection.

A total of 330 articles were identified *via* PubMed search, and five articles were retrieved from other sources. Of these 335 papers, 114 were reviews and editorials, 22 case studies, two study protocols, and two clinical guidelines. Another 152 were deemed irrelevant by consensus between two investigators (NDK and OVS) if articles did not pertain to human subjects, lacked VKA treatment or effect estimates, or had no standardized method of detecting and quantifying calcification. After a full-text review of the remaining 43 articles, an additional seven were excluded due to missing data (*n* = 1), not matching the inclusion criteria (*n* = 4), being a secondary analysis of an already included study (*n* = 2), or an ongoing study (*n* = 1, Figure 1). Thus, 35 studies and 45,757 participants were included in the analysis, and 6,251 were treated with VKA (27–61). Of these, three studies were randomized trials (4 independent analysis cohorts, 333 patients, 169 VKA users) (29, 32, 39), and 32 observational studies (38 cohorts; 45,424 participants; 6,082 VKA users) (27, 28, 30, 31, 33–38, 40–61). Thirteen studies investigated the effects of VKA on coronary artery calcification (15 cohorts, 23,768 participants, 2,625 received VKA) (27, 28, 30, 31, 33–38). Sixteen studies that reported extra-coronary (any artery but coronary) calcification included 18 cohorts, 4,740 participants, and 1,595 patients treated with VKA (40–54). Finally, nine studies investigated the effects of VKA treatment on aortic valve calcification (9 cohorts; 17,161 participants; 1,987 VKA-treated patients) (31, 49, 55–61).

Characteristics of the included studies are shown in Table 1. Studies were published between 2005 and 2022. Twenty studies were conducted in Europe (29–31, 34–36, 38, 41–43, 46–51, 53, 55, 57–59), thirteen in North America (27, 28, 32, 33, 37, 39, 40, 44, 45, 52, 56, 58, 60), and two in Asia (54, 61). As stated above, we identified three randomized trials (29, 32, 39), one meta-analysis of patient-level data from eight randomized trials (27), twenty one retrospective cohort studies (30, 31, 34–38, 40–42, 45, 48–52, 54, 55, 57, 59, 60), one prospective cohort (61), and nine cross-sectional studies (28, 33, 43, 44, 46, 47, 53, 56, 58). The sample size ranged from 37 to 17,254 participants. The median sample size was 207, interquartile range (IQR) of 108 to 387 patients. The age of participants was 66.2 ± 3.6 years (weighted mean ± SD); 77% (IQR 72–95%) of participants were males. The weighted median duration of VKA treatment in 33 groups of prospectively or retrospectively followed patients was 2.3 years (IQR 1.2–4.0) (27, 29–32, 34–42, 45, 48–52, 54, 55, 57, 59–61). Nine study cohorts were cross-sectional with an unspecified duration of treatment (28, 33, 43, 44, 46, 47, 53, 56, 58). The medical history of the patients included coronary artery disease (CAD) (27, 32, 39), chronic kidney disease CKD (including patients with ESRD) (29, 40–43, 46, 47, 53, 55), calcific aortic valve disease or aortic stenosis (CAVD/AS) (28, 56, 57, 60), atrial fibrillation (AF/NVAF) (28, 29, 32, 34, 38, 39, 49, 54, 55, 58, 61), metallic prosthetic valves (36), lower limb amputation (44), carotid atherectomy (48), non-traumatic cerebral hemorrhage (50) or underwent cardiac CT (33, 35) or mammography (40, 52) tests for diagnostic or screening purposes. Two studies included the general population from health registries (30, 59). For the remaining two studies, patients' medical history was not specified (37, 51).

**Table 1:** Characteristics of the included studies.

**References**	**Country**	**Study design**	**Sample size VKA - Y/N**	**Patients characteristics**	**Age VKA - Y/N**	**% Males VKA - Y/N**	**Treatment or exposure**	**Duration**	**Outcome**	**Assessment**	**Effect size estimate**	**Parameter estimate - methods and adjustments**
**Coronary artery calcification**												
Andrews et al. (27)	United States	Patient-level meta-analysis	171/4129	CAD	62/58	80/72	Warfarin/no exposure	18–24 mo	CAC index	IVUS	OR for an increase of calcium index	Multivariable regression adjusted for age, BMI, rank-transformed baseline calcium index, baseline percent atheroma volume (PAV), change in PAV, and last observation of creatinine, clinical trial, treatment, study time duration
Chaikriangkrai et al. (28)	United States	Cross-sectional	154/706	AF, no CAD	63 (all)	65 (all)	Warfarin/no exposure	n/a	CAC score >0	CT	OR for calcification	Univariable regression; unadjusted
De Vriese et al. (29)	Belgium	Prospective randomized	44/46	ESRD, NVAF	80/80	57/76	VKA/rivaroxaban	18 mo	CAC	CT	Change from baseline	Kruskal–Wallis test
Hasific et al. (30)	Denmark	Retrospective cohort	1748/15506	no CAD	67 (all)	75 (all)	Warfarin time-updated exposure	14 mo	CAC score	CT	OR for higher CAC category per year	Multivariable regression adjusted for age, gender, smoking, BMI, diabetes, hypertension, hypercholesterolemia, family history of CVD, eGFR, NOAC treatment duration
Koos et al. (31)	Germany	Retrospective cohort	23/63	CAVD	71 (all)	62 (all)	VKA/no exposure	7.3 yrs	CAC score	CT	CAC score mean, SD	Student's *t*-test
Lee et al. (32)	United States	Prospective randomized	51/46	NVAF, CAC >10	60/63	77/65	Warfarin/rivaroxaban	12 mo	Calcified plaque volume	CCTA	Regression coefficient	Multivariable regression adjusted for age, gender, BMI, hypertension, diabetes, dyslipidemia, baseline LDL cholesterol, current smoking, family history, statin use, and baseline normalized plaque volume
Palaniswamy et al. (33)	United States	Cross-sectional	28/205	cardiac CT testing	67/63	71/56	Warfarin/no exposure	n/a	CAC score	CT	Incidence of CAC score >100	Prevalence
Plank et al. (34)	Austria	Retrospective cohort	101/101	NVAF, no CAD	60/60	73/70	VKA/no exposure	20 mo	CAC score	CT	CAC score mean, SD	ANOVA. Cohorts were matched according to the propensity score for age, male sex, hypertension, hyperlipidemia, diabetes, family history of premature cardiac death, smoking, BMI
Schurgers et al. (35)	Netherlands	Retrospective cohort	44/44	diagnostic cardiac CT	58/58	66/66	VKA/no exposure	2.5 mo	CAC score	CT	CAC score mean, SD	ANOVA. Patients were matched according to the Framingham risk score (FRS)
Schurgers et al. (35)	Netherlands	Retrospective cohort	44/44	diagnostic cardiac CT	60/60	61/61	VKA/no exposure	19 mo	CAC score	CT	CAC score mean, SD	ANOVA. Patients were matched according to FRS
Schurgers et al. (35)	Netherlands	Retrospective cohort	45/45	diagnostic cardiac CT	64/59	78/78	VKA/no exposure	7.2 yrs	CAC score	CT	CAC score mean, SD	ANOVA. Patients were matched according to FRS
Unlu et al. (36)	Turkey	Retrospective cohort	43/65	metallic prosthetic valve	57/54	35/39	VKA/no exposure	15 yrs	CAC score	CT	CAC score mean, SD	Mann–Whitney *U*-test. Patients were matched according to atherosclerotic risk factors
Villines et al. (37)	United States	Retrospective cohort	28/31	no CAD	73/64	68/68	5.9 yrs/1 mo warfarin	5.9 yrs	CAC score	CT	CAC score median, IQR, Min, Max	ANOVA
Weijs et al. (38)	Netherlands	Retrospective cohort	71/86	AF, no CAD	58/56	79/62	VKA time-updated exposure	3.8 yrs	CAC score	CT	OR for an increase of CAC category/ year	Multivariable regression adjusted for age, left atrium diameter, use of statins and ACE inhibitors
Win et al. (39)	United States	Prospective randomized	30/26	NVAF	55/60	80/58	Warfarin/apixaban	12 mo	Calcified plaque volume	CCTA	Regression coefficient	Multivariable regression adjusted for age, gender, BMI, hypertension, diabetes, dyslipidemia, smoking, family history, prior percutaneous coronary intervention, coronary bypass surgery, aspirin use, statin use, and baseline plaque volume
**Extra-coronary arterial calcification**												
Alappan et al. (40)	United States	Retrospective cohort	35/57	BAC, no CKD	76/74	0/0	Warfarin/no exposure	8.3 yrs	BAC rate (mm/yr)	Mammogram	Log-modulus BAC rate per year	Kruskal-Wallis test
Alappan et al. (40)	United States	Retrospective cohort	29/95	BAC, CKD	79/76	0/0	Warfarin/no exposure	4.1 yrs	BAC rate (mm/yr)	Mammogram	Log-modulus BAC rate per year	Kruskal-Wallis test
Alappan et al. (40)	United States	Retrospective cohort	14/36	BAC, ESRD	61/60	0/0	Warfarin/no exposure	3.9 yrs	BAC rate (mm/yr)	Mammogram	Log-modulus BAC rate per year	Kruskal-Wallis test
De Vriese et al. (29)	Belgium	Prospective randomized	44/46	ESRD, NVAF	80/80	57/76	VKA/rivaroxaban	18 mo	TA calcification score	CT	Change from baseline	Kruskal–Wallis test
Eren-Sadioglu et al. (41)	Turkey	Retrospective cohort	32/44	ESRD	68/65	56/50	Warfarin/no exposure	5.5 yrs	AA Kauppila score (62) >6	X-ray	OR of Kauppila score of >6	Multivariable regression adjusted for age, PTH, serum calcium, serum phosphorus; dialysis vintage; patients were matched according to age, sex, comorbidities, dialysis vintage, and dialysis center.
Fusaro et al. (42)	Italy	Retrospective cohort	46/341	ESRD	70/63	59/63	Warfarin/no exposure	4.2 yrs	AA calcification grade	X-ray	OR of calcification	Multivariable regression adjusted for age, angina, AF, PPI use, total BGP
Fusaro et al. (43)	Italy	Cross-sectional	101/213	ESRD	72 (all)	63 (all)	VKA/no exposure	n/a	AA calcification score (63)	X-ray	OR of severe calcification	Multivariable regression adjusted for age, sex, dialysis vintage, HF, PAD, stroke, plasma vitamin D, vertebral fractures
Han et al. (44)	United States	Cross-sectional	29/79	Lower limb amputation	64 (all)	51 (all)	Warfarin/no exposure	n/a	Lower extremity calcification	Histopathology	Incidence of calcification	Fisher's exact test
Han and O'Neill. (45)	United States	Retrospective cohort	430/430	no ESRD	67/67	41/41	Warfarin time-updated exposure	9.8 mo	Lower extremity calcification	X-ray	OR of calcification per log days of warfarin	Multivariable regression adjusted for age, diabetes status, sex, duration of warfarin use, serum creatinine, radiograph type
Jean et al. (46)	France	Cross-sectional	32/129	ESRD	67 (all)	55 (all)	Warfarin/no exposure	n/a	AA, IA, FA calcification score	X-ray	OR of calcification score 2 or 3	Multivariable regression adjusted for age, sex, FGF-23, diabetes, smoking, peripheral vascular disease, CAD, albumin, OPG, CRP
Jean et al. (47)	France	Cross-sectional	44/163	ESRD	70 (all)	57 (all)	Warfarin/no exposure	n/a	AA Kauppila score (86) >7	X-ray	OR of Kauppila score >7	Prevalence
Nuotio et al. (48)	Finland	Retrospective cohort	82/418	carotid atherectomy	75/69	73/67	Warfarin/no exposure	19 mo	CCA calcification, Y/N	CT	OR of calcification	Multivariable regression adjusted for age, sex, and smoking
Peeters et al. (49)	Netherlands	Retrospective cohort	71/86	AF, no prior CAD	58/56	80/62	VKA/no exposure	2.3 yrs	AscA calcification score	CT	OR of calcification	Multivariate regression adjusted for the propensity score for age, sex, BMI, systolic BP, family history of MI, hyperlipidemia, blood glucose, LA dimension
Peeters et al. (50)	Netherlands	Retrospective cohort	77/299	Non-traumatic cerebral hemorrhage	78/70	54/53	VKA/no exposure	2.9 yrs	ICA calcification score	CT	OR of high calcification score	Multivariable regression adjusted for age, sex, hypertension, hypercholesterolemia, and diabetes
Rennenberg et al. (51)	Netherlands	Retrospective cohort	19/18	Risk of thrombosis, no prior CAD	48/56	79/50	Coumarin/no exposure	13 yrs	FA calcification, Y/N	X-ray	Regression coefficient	Multivariable regression adjusted for age, smoking, BMI, and triglycerides
Tantisattamo et al. (52)	United States	Retrospective cohort	451/451	Mammography	68/68	0 (all)	VKA time-updated exposure	4.6 yrs	BAC, Y/N	Mammogram	OR of calcification per year	Multivariable regression adjusted for age, sex, diabetes, indications for warfarin, warfarin-free duration, serum creatinine, serum calcium, and statin use
Van Berkel et al. (53)	Belgium	Cross-sectional	24/286	CKD, ESRD, renal Tx	59 (all)	0 (all)	VKA/no exposure	n.a	BAC Y/N	Mammogram	BAC, Y/N	Prevalence
Wei et al. (54)	China	Retrospective cohort	79	NVAF	64 (all)	51 (all)	Warfarin time-updated exposure	5 mo	AA calcification score	CT	OR of score change by 1 SD per year	Multivariable regression adjusted for age, BMI, smoking, ALP, LDL cholesterol, CRP, warfarin dose, and INR
**Aortic valve calcification**												
Di Lullo et al. (55)	Italy	Retrospective cohort	100/247	NVAF, CKD	67/66	58/54	Warfarin/rivaroxaban	16 mo	AVC, change from baseline	US	Regression coefficient	Multivariable regression adjusted for baseline aortic calcification, systolic BP, eGFR, diabetes, glycated hemoglobin, PTH
Ing et al. (56)	United States	Cross-sectional	11/184	AS	71 (all)	78 (all)	VKA/no exposure	n/a	AV ossification Y/N	Histopathology	OR of presence of ossification	Multivariable regression adjusted for sex, sex, diabetes
Koos et al. (31)	Germany	Retrospective cohort	23/63	CAVD	71 (all)	62 (all)	VKA/no exposure	7.3 yrs	Agatston score	CT	Mean, SD	Student's *t*-test
Koos et al. (57)	Germany	Retrospective cohort	27/164	CAVD	71 (all)	71 (all)	VKA/no exposure	> 4 yrs	AVC score	CT	F statistics	ANCOVA adjusted for sex, age, sex, BMI, diabetes, smoking, hypertension, hypercholesterolemia, eGFR, use of the beta-blockers, ACE inhibitors, diuretics, cholesterol-lowering medications, thyroid hormones, and antidepressants
Lerner et al. (58)	United States	Cross- sectional	725/430	NVAF	74/74	61/61	Warfarin/no exposure	n/a	AV calcification, Y/N	US	OR of calcification	Multivariable regression adjusted for age, sex, race, eGFR, serum ALP, calcium, phosphate, and calcium-phosphate product
Peeters et al. (49)	Netherlands	Retrospective cohort	71/86	AF, no CAD	58/56	80/62	VKA/no exposure	2.3 yrs	AVC score	CT	OR of calcification	Multivariable regression adjusted for the propensity score for age, sex, BMI, systolic BP, family history of MI, hyperlipidemia, blood glucose, and LA dimension
Sonderskov et al. (59)	Denmark	Retrospective cohort	873/13,731	general population	67 (all)	95 (all)	VKA/no exposure	2.5 yrs	AVC score (arbitrary)	CT	REC per year	Multivariable negative binomial regression adjusted for age, sex, hypertension, diabetes mellitus, creatinine clearance, statins, and sq root AVC score at baseline
Tastet et al. (60)	Canada	Retrospective cohort	35/166	AS (mild)	79/65	71 (all)	Warfarin/no exposure	24 mo	AVC Agatston score rate (100 pe year)	CT	Regression coefficient	Multivariable regression adjusted for gender, age, BMI, diabetes mellitus, hypertension, dyslipidemia, smoking status, known CVD, family history of CVD, and eGFR
Yamamoto et al. (61)	Japan	Prospective cohort	122/101	NVAF	70/69	79/68	Warfarin/no exposure	4 yrs	AV number of calcified leaflets	US	Incidence of progression	Incidence

AA, abdominal aorta; ACE, angiotensin-converting enzyme; AF, atrial fibrillation; ALP, alkaline phosphatase; ANCOVA, analysis of covariance; ANOVA, analysis of variance; AS, aortic stenosis; AscA, ascending aorta; AV, aortic valve; AVC, aortic valve calcification; BAC, breast artery calcification; BGP, bone Gla protein; BMI, body mass index; BP, blood pressure; CAC, coronary artery calcium; CAD, coronary artery disease; CAVD, calcific aortic valve disease; CCA, common carotid artery; CCTA, cardiac computed tomography angiography; CKD, chronic kidney disease; CRP, C-reactive protein; CT, computed tomography, CVD, cardiovascular disease; eGFR, estimated glomerular filtration rate; ESRD, end-stage renal disease; FA, femoral artery; FGF-23, fibroblast growth factor 23; FRS, Framingham risk score; HF, heart failure; IA, iliac artery; ICA, internal carotid artery; INR, international normalized ratio; IQR, interquartile range; IVUS, intravascular ultrasound; LA, left atrium; LDL, low-density lipoprotein; MI, myocardial infarction; NOAC, non-VKA oral anticoagulants (DOAC, direct oral anticoagulants); NVAF, non-valvular atrial fibrillation; OPG, osteoprotegerin; OR, odds ratio; PAD, peripheral arterial disease; PAV, percent atheroma volume; PPI, proton pump inhibitor; PTH, parathyroid hormone; REC, ratio of expected counts; SD, standard deviation; TA, thoracic aorta; US, ultrasound; VKA, vitamin K antagonist.

### Quality assessment

Among the three randomized trials, one “per-protocol” trial (29) had a high risk of bias due to missing outcome data, whereas two other “intention-to-treat” studies had concerns regarding missing outcome data (32) and selective reporting (32, 39) (Supplementary Table 2). Observational studies were assessed for the risk of bias on a 9-point Newcastle-Ottawa quality scale. The majority of studies were of at least moderate quality (27, 28, 30, 31, 34–38, 40–43, 45, 46, 48–59, 61) [median score 7 (IQR 5-9)], except five studies in which the risk of bias was considered low to moderate on the Newcastle-Ottawa quality scale (31, 33, 44, 47, 53) (Supplementary Table 3).

### Effects of VKA use on the coronary, extra-coronary, and aortic valve calcification

**Figure 2:**
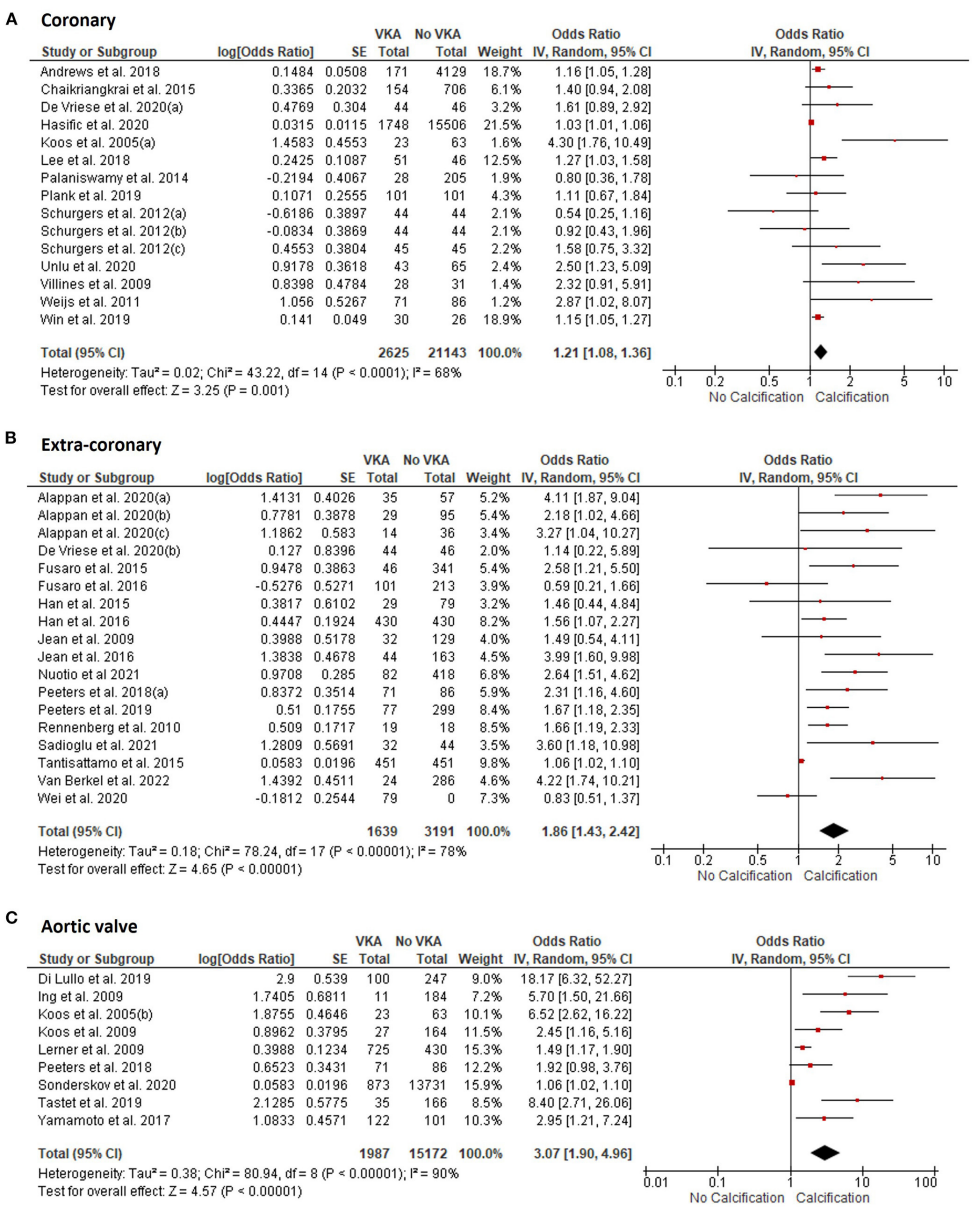
Meta-analysis of vascular and valvular calcification studies in VKA-treated patients. **(A)** coronary artery calcification; **(B)** extra-coronary calcification; **(C)** aortic valve calcification studies.

VKA use was associated with increased vascular and valvular calcification. The OR for the coronary artery calcification in VKA-treated patients was 1.21 (95% CI 1.08, 1.36), *p* = 0.001 compared to patients not treated with VKA (Figure 2A). VKA use was also associated with extra-coronary vascular calcification in the aorta, carotid arteries, breast arteries, and arteries of lower extremities [OR 1.86 (1.43, 2.42), *p* < 0.00001, Figure 2B]. Furthermore, we found an association between VKA use and aortic valve calcification [OR 3.07 (1.90, 4.96), *p* < 0.00001, Figure 2C]. Between-study heterogeneity was significant at *I*^2^ of 69, 78, and 90% in the coronary (*n* = 15), extra-coronary vascular (*n* = 18), and aortic valve studies (*n* = 9), respectively.

### Publication bias

**Figure 3:**
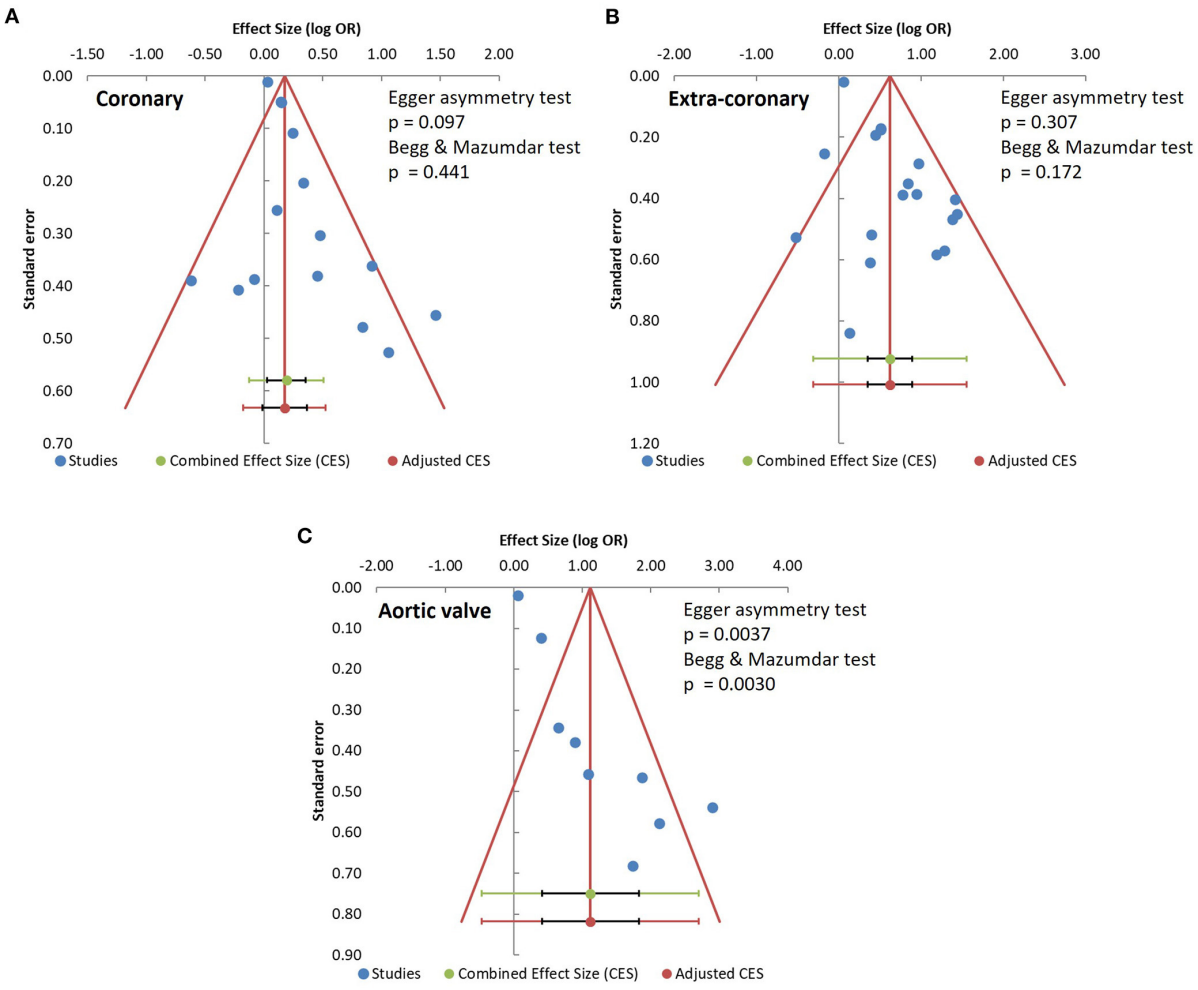
Analysis of publication bias. **(A)** coronary artery calcification; **(B)** extra-coronary calcification; **(C)** aortic valve calcification studies.

We constructed funnel plots of the effect sizes against their standard errors [log (OR), SE] and examined them using the Egger funnel plot asymmetry test and Begg & Mazumdar rank correlation test to evaluate the risks of publication bias. No significant risks of publication bias were found among the coronary artery calcification studies (Egger *p* = 0.097, Begg & Mazumdar *p* = 0.441) or the extra-coronary calcification studies (Egger *p* = 0.307, Begg & Mazumdar *p* = 0.172, Figures 3A,B). However, the risk of publication bias was significant in the studies of aortic valve calcification (Egger *p* = 0.0037, Begg & Mazumdar *p* = 0.0030, Figure 3C).

### Meta-regression and subgroup analysis

**Figure 4:**
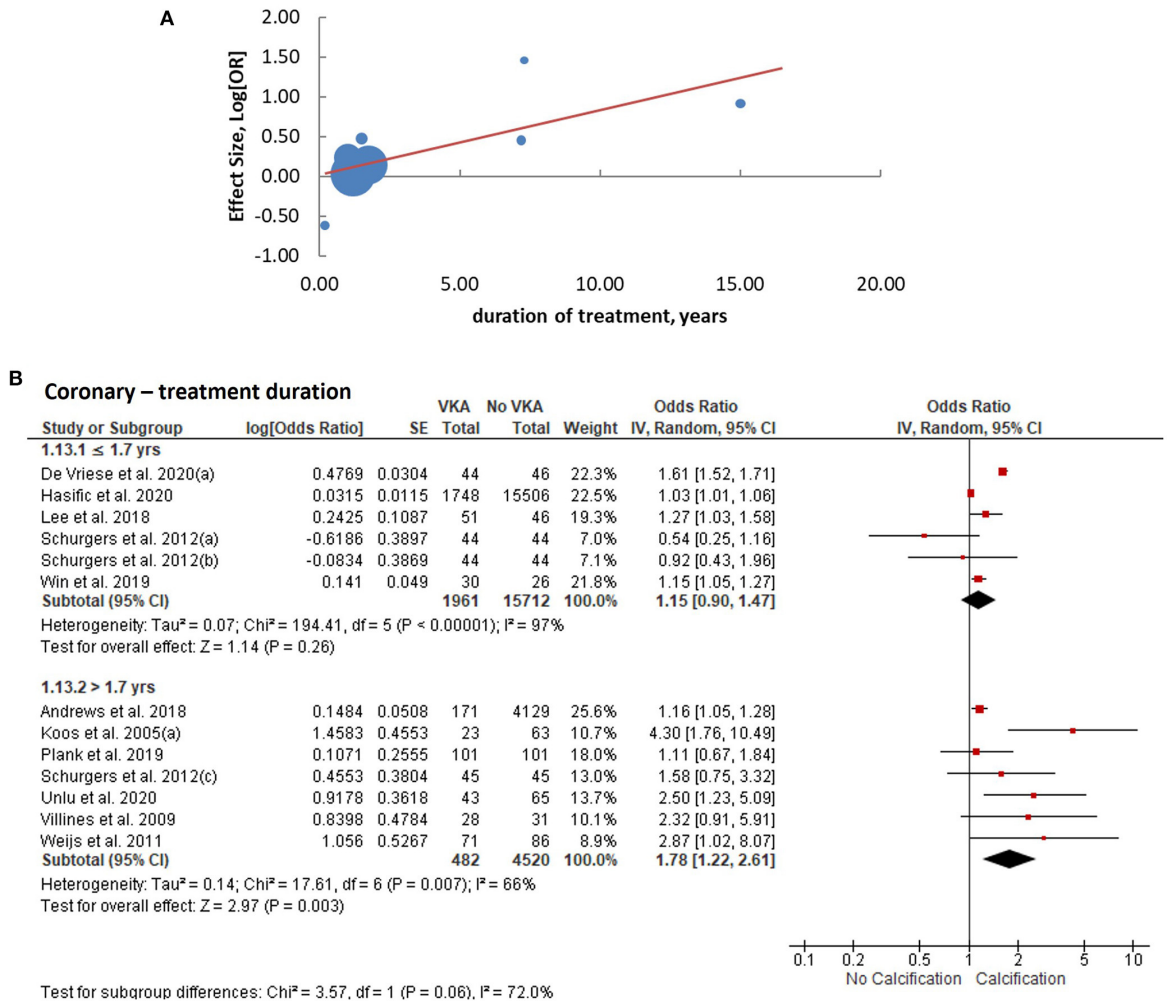
Analysis of coronary arterial calcification according to the duration of VKA treatment. **(A)** meta-regression of the effect sizes—less or equal vs. more than the median duration; **(B)** subgroup analysis based on the duration of treatment.

**Figure 5:**
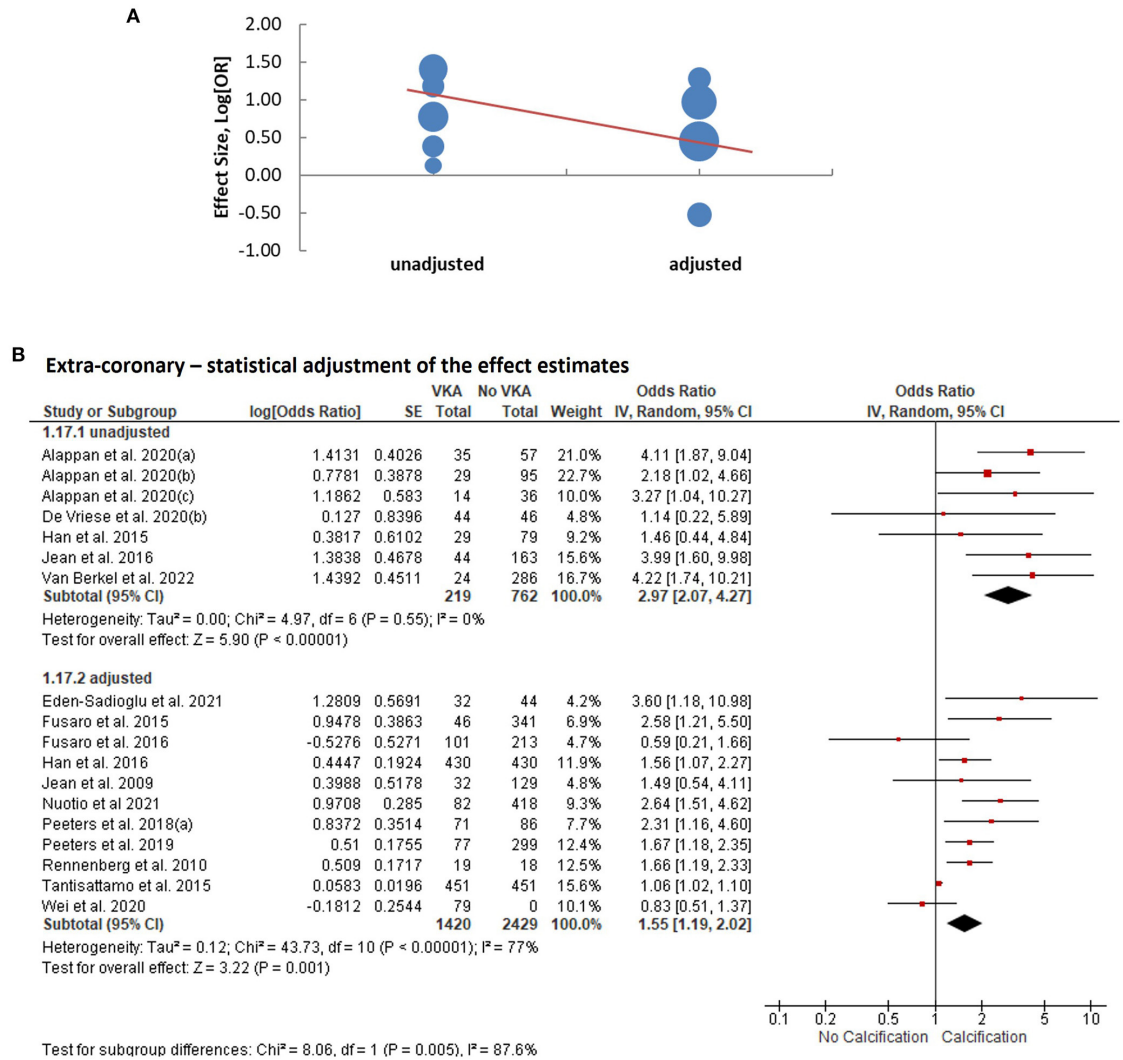
Analysis of extra-coronary arterial calcification according to statistical adjustment for confounding variables. **(A)** Meta-regression of the effect sizes—adjusted vs. unadjusted. **(B)** Subgroup analysis based on whether or not statistical adjustments were applied to calculate the effect estimates.

We performed meta-regression analyses to identify potential effect modifiers. We calculated univariate random-effects regressions to assess the effects of the year of publication, geographic region (continent), study design, sample size, patient characteristics, median age, ratio of participants by sex, duration of VKA treatment, calcium imaging modality, and whether or not the effect estimates were reported adjusted for the confounders (Table 1). The estimates of coronary artery calcification were influenced by three explanatory variables, the year of publication [*B* regression coefficient of −0.04 (95% CI: −0.08, 0.00), *p* = 0.035]; the gender ratio expressed as a percent of male participants [*B* = −0.01 (−0.03, 0.00), *p* = 0.039]; and the duration of VKA treatment [*B* = 0.08 (0.03, 0.13), *p* = 0.0005, Table 2; Figure 4A]. The effects on the extra-coronary vascular calcification were modified by whether or not the reported estimates were adjusted or not adjusted for the confounders [*B* = −0.63 (−1.19, −0.08), *p* = 0.016, Table 2; Figure 5A]. Although the number of the aortic valve studies was low (*n* = 9) and suffered from a significant risk of publication bias, we performed a meta-regression analysis and found that the effect estimates were potentially modified by the sample size [*B* = −0.32 (−2.35, −0.04), *p* = 0.009, Table 2].

**Table 2:** Meta-regression analysis of potential effect moderators.

**Effect moderator**	**Coronary**		**Extra-coronary**		**Aortic valve**	
	**B coefficient (95% CI)**	***P*-value**	**B coefficient (95% CI)**	***P*-value**	**B coefficient (95% CI)**	***P*-value**
Publication year	−0.04 (−0.08, 0.00)	0.035	0.05 (−0.03, 0.12)	0.183	0.00 (−0.14, 0.14)	0.953
Geographic region	−0.03 (−0.33, 0.26)	0.803	−0.18 (−0.70, 0.34)	0.458	0.11 (−1.51, 1.73)	0.873
Study design	−0.01 (−0.24, 0.22)	0.908	0.11 (−0.53, 0.75)	0.711	−0.35 (−2.35, 1.65)	0.688
Sample size, log (*N*)	−0.04 (−0.10, 0.02)	0.164	−0.08 (−0.35, 0.19)	0.520	−0.32 (−0.60, −0.04)	0.009
Patient characteristics	−0.22 (−0.54, 0.11)	0.153	0.33 (−0.24, 0.91)	0.216	0.82 (−0.18, 1.82)	0.059
Age, years	0.01 (−0.01, 0.03)	0.316	0.00 (−0.03, 0.03)	0.953	0.04 (−0.09, 0.17)	0.459
Sex ratio (% males)^a^	−0.01 (−0.03, 0.00)	0.039	0.01 (−0.01, 0.03)	0.279	−0.04 (−0.11, 0.02)	0.111
Duration of treatment, years^b^	0.08 (0.03, 0.13)	0.0005	0.02 (−0.06, 0.09)	0.657	0.01 (−0.40, 0.42)	0.947
Imaging modality^c^	−0.31 (−1.93, 1.31)	0.653	−0.22 (−0.86, 0.43)	0.476	−0.31 (−1.93, 1.31)	0.653
Adjustment for confounders	−0.18 (−0.46, 0.09)	0.154	−0.63 (−1.19, −0.08)	0.016	−0.48 (−1.80, 0.84)	0.401

a excluding BAC studies;

b excluding cross-sectional studies;

c excluding histopathology.

We consequently performed subgroup analyses comparing the top and bottom half of the studies with respect to each of the identified modifiers (publication year, sex ratio, duration of VKA treatment, and statistical adjustment). We found that the effect of VKA duration on the coronary artery calcification was borderline significant when comparing studies of a longer duration (>1.7 years) and studies of ≤ 1.7 years duration (groups difference test *I*^2^ = 72%, *p* = 0.06; Table 3; Figure 4B). We also found that, as predicted by meta-regression, the estimates of VKA effects of the extra-coronary calcification were modified by whether or not the reported models were adjusted for plausible confounders (*I*^2^ = 79%, *p* = 0.005, Table 3; Figure 5B).

**Table 3:** Subgroup analysis by study design.

**Moderator**	**Subgroup**	**Number of studies**	**Patients—VKA (Y/N)**	**OR (95% CI)**	***P*-value**	***I* ^2^**	**Test for subgroup differences *p*-value**
**Coronary**							
Publication year	Before 2016	8	437/1,224	1.43 (0.93, 2.18)	0.10	61%	0.63
	2017–2022	7	2,188/19,919	1.27 (1.04, 1.55)	0.02	97%	
Sample size	**≤**97	8	309/345	1.31 (1.03, 1.66)	0.03	58%	0.42
	>97	7	2,316/20,798	1.16 (1.01, 1.35)	0.04	65%	
Sex ratio (%males)	**≤**71%	8	481/1,274	1.33 (0.92, 1.92)	0.13	62%	0.48
	>71%	7	2,144/19,869	1.16 (1.04, 1.28)	0.006	70%	
Duration^a^	**≤**1.7 yrs	7	1,961/15,712	1.15 (0.90, 1.47)	0.26	97%	0.06
	>1.7 yrs	6	482/4,520	1.78 (1.22, 2.61)	0.003	66%	
Adjustment for confounders	Unadjusted	10	554/1,350	1.40 (1.02, 1.93)	0.04	55%	0.21
	Adjusted	5	2,071/19,793	1.14 (1.03, 1.26)	0.01	76%	
**Extra-coronary**							
Publication year	Before 2019	9	1,223/1,910	1.59 (1.16, 2.19)	0.04	74%	0.21
	2019–2022	9	416/1,281	2.21 (1.48, 3.29)	<0.0001	63%	
Sample size	**≤**159	9	352/461	1.93 (1.33, 2.80)	0.0005	53%	0.80
	>159	9	1,287/2,730	1.80 (1.26, 2.58)	0.001	82%	
Sex ratio (%males)^b^	**≤**56	7	703/1,267	1.65 (1.16, 2.36)	0.006	55%	0.46
	>56	7	407/1,285	2.00 (1.40, 2.86)	0.0001	44%	
Duration^a^	**≤**4.1 yrs	7	797/1,315	1.68 (1.22, 2.30)	0.001	52%	0.50
	>4.1 yrs	6	612/1006	2.04 (1.28, 3.25)	0.003	84%	
Adjustment for confounders	Unadjusted	7	219/762	2.97 (2.07, 4.27)	<0.00001	0%	0.005
	Adjusted	11	1,420/2,429	1.55 (1.19, 2.02)	0.001	77%	
**Aortic valve**							
Publication year	Before 2018	5	908/942	2.93 (1.57, 5.48)	0.0008	73%	0.70
	2018–2022	4	1,079/14,230	3.82 (1.14, 12.87)	0.03	93%	
Sample size	**≤**201	5	167/663	3.83 (2.11, 6.95)	<0.00001	53%	0.20
	>201	4	1,820/14,509	2.24 (1.26, 3.99)	0.006	92%	
Sex ratio (%males)	**≤**71%	5	910/1,070	4.76 (1.82, 12.43)	0.001	89%	0.16
	>71%	4	1,077/14,102	2.03 (1.01, 4.08)	0.03	79%	
Duration^a^	**≤**2.5 yrs	4	1079/14,230	3.82 (1.14, 12.87)	0.03	93%	0.89
	>2.5 yrs	3	172/328	3.47 (1.95, 6.19)	<0.0001	29%	
Adjustment for confounders	Unadjusted	2	145/164	4.37 (2.01, 9.50)	0.0002	32%	0.31
	Adjusted	7	1,842/15,008	2.72 (1.64, 4.50)	0.0001	90%	

a excluding cross-sectional studies;

b excluding BAC studies.

### Sensitivity analysis

To explore the influence of any single study on the overall effect sizes, we excluded studies from the corresponding analyses, one at a time. No significant effects of any individual study on the effect sizes of VKA were observed (Supplementary Table 4).

## Discussion

We present the first meta-analysis of the effects of vitamin K antagonists on vascular and valvular calcification. Our results confirmed a strong association between VKA use and vascular calcification in the absence of significant risks of publication bias. We also found evidence of a positive association between VKA use and aortic valve calcification; however, due to a smaller number of studies and evidence of publication bias, the confidence in this finding is low.

A large number of good-quality observational studies of the effects of VKA on vascular calcification have been published. These studies assessed calcification in thousands of VKA-treated patients. Improved imaging modalities and the use of calcium imaging in diagnostics studies allowed for the analysis of larger cohorts of patients with greater precision. Several authors took a propensity matching approach to eliminate the potential confounders; others used other comprehensive statistical methods to minimize the effects of confounding variables on calcification estimates. The quality of publications on VKA use and vascular calcification is also supported by a lack of significant risk of publication bias and the fact that we could detect that duration of treatment as a modifier of the effect estimates. Lastly, a recent introduction of a new class of non-vitamin K oral anticoagulants, NOAC, allowed for the analysis of calcification in the first head-to-head randomized trials with VKA, although each of the three randomized trials assessed <100 patients so far.

Of 45,000 participants included in this meta-analysis, 99.5% were from observational studies. Therefore, it is important to recognize limitations inherent to observational study designs, such as the difficulty of establishing a cause-and-effect relationship. We also observed that the magnitude of effect estimates was modified by several experimental parameters, including sample size, gender ratio, and adjustments for confounding variables. In addition, we found that studies of VKA in valvular calcification suffered from a significant risk of publication bias, limiting our confidence in that association.

For decades, warfarin, a commonly used VKA, has been a standard and effective treatment option for patients requiring anticoagulation. Early studies in the 1980 s found an association between dystrophic calcification and warfarin in an animal model examining bioprosthetic aortic valves (64). Since the early 1990 s, it has been known that warfarin is associated with soft tissue calcification, such as skin calcinosis and tracheobronchial calcification (65, 66). In 2005, Koos et al. documented for the first time the effects of warfarin on the coronary artery and aortic valve calcification (31).

Cardiovascular calcification presents several morphologically distinct forms, including the intimal, medial, and heart valve calcification. Coronary arteries are primarily affected by atherosclerotic intimal calcification, whereas the peripheral arteries and aorta show different degrees of medial and intimal involvement. Despite the anatomical difference, many significant correlations exist between calcification burdens at different vascular sites, suggesting a common mechanism and the influence of systemic factors. Thus, calcification in the abdominal aorta, breast and the arteries of the extremities artery correlates with coronary artery calcification (67–69). An independent association between aortic valve calcification and the severity of coronary artery calcification has also been reported (70).

Calcification is initiated by osteogenic transdifferentiation of vascular cells (71). Transdifferentiated cells secrete mineralizing matrix vesicles that serve as nucleating sites for extracellular calcium deposition (72–74). Osteogenic transdifferentiation is preceded by inflammation, as has been shown in a longitudinal study of patients' coronary artery calcification employing positron emission tomography (PET) combined with CT (75). Active inflammation and microcalcification detected by specific PET were also shown to co-exist in patients with peripheral arterial or calcific aortic valve disease (76, 77). MGP interferes with both the osteogenic cell transformation and physicochemical process of biomineralization (78). VKA reduces gamma-carboxylation leaving MGP in the inactive state.

Calcification can negatively affect the clinical course of cardiovascular disease in several ways, by increasing arterial stiffness, stability of atherosclerosis plaques, and, in the context of calcific aortic valve stenosis, reducing the opening of the valves. Arterial stiffness promotes microcirculatory damage by increasing the transmission of pressure pulsatility (79). Numerous studies have shown that arterial stiffness predicts cardiovascular outcomes after adjustments for conventional risk factors (80–82). Calcification also changes the composition of atherosclerotic plaque. An earlier study of culprit plaques' characteristics documented that surface erosion over a calcified nodule has likely precipitated an acute ischemic coronary event and death (83). The later studies using ^18^F-sodium fluoride positron emission tomography (^18^F-NaF PET), capable of detecting micro-calcification invisible to other imaging technologies, demonstrated that micro-calcification is associated with high-risk plaque features (84). Furthermore, aortic valve calcification and the rate of progression of calcification are strong predictors of aortic valve stenosis outcomes (85, 86). Thus, the association between VKA use and cardiovascular calcification is concerning because it might worsen the course of vascular or valvular disease.

It was suggested that warfarin-induced calcification could result in adverse clinical outcomes (87). One study included in this meta-analysis demonstrated that warfarin had a significant hazard ratio of 1.97 for the overall mortality in hemodialysis patients independent of the confounder variables, age, atrial fibrillation, and diabetes. Furthermore, adjustment for vascular calcification reduced the strength of this association, suggesting that warfarin-induced calcification might have contributed to mortality (42).

In conclusion, our meta-analysis demonstrates that VKA use is associated with vascular calcification. Thus, vascular calcification can be considered a side effect of VKA. However, the clinical significance of VKA-induced calcification and the risk benefits of VKA therapy requires further evaluation.

## Data availability statement

The original contributions presented in the study are included in the article/Supplementary material, further inquiries can be directed to the corresponding author/s.

## Author contributions

NDK and OVS conceived, designed the review, analyzed, interpreted the results, and edited the manuscript. NDK and MS performed the literature search and screened the data. NDK and DK extracted the data. OVS verified the extracted data. NDK wrote the first draft. All authors contributed to the article and approved the submitted version.

## Funding

This work was supported by the National Heart, Lung, and Blood Institute of the National Institutes of Health under award number R01HL149864 (OVS) and the NYITCOM Academic Medicine Scholar Program (NDK).

## Conflict of interest

The authors declare that the research was conducted in the absence of any commercial or financial relationships that could be construed as a potential conflict of interest.

## Publisher's note

All claims expressed in this article are solely those of the authors and do not necessarily represent those of their affiliated organizations, or those of the publisher, the editors and the reviewers. Any product that may be evaluated in this article, or claim that may be made by its manufacturer, is not guaranteed or endorsed by the publisher.

## References

[1] Shekar C Budoff M. Calcification of the heart: mechanisms and therapeutic avenues. Exp Rev Cardiovasc Therapy. (2018) 16:527–36. 10.1080/14779072.2018.1484282 29860888 PMC6309454

[2] Agatston AS Janowitz WR Hildner FJ Zusmer NR Viamonte M Jr Detrano R. Quantification of coronary artery calcium using ultrafast computed tomography. J Am Coll Cardiol. (1990) 15:827–32. 10.1016/0735-1097(90)90282-T 2407762

[3] Breen JF Sheedy PF 2nd Schwartz RS Stanson AW Kaufmann RB Moll PP . Coronary artery calcification detected with ultrafast CT as an indication of coronary artery disease. Radiology. (1992) 185:435–9. 10.1148/radiology.185.2.1410350 1410350

[4] Margolis JR Chen JT Kong Y Peter RH Behar VS Kisslo JA. The diagnostic and prognostic significance of coronary artery calcification. A report of 800 cases. Radiology. (1980) 137:609–16. 10.1148/radiology.137.3.7444045 7444045

[5] Sugiyama T Yamamoto E Fracassi F Lee H Yonetsu T Kakuta T . Calcified plaques in patients with acute coronary syndromes. JACC Cardiovasc Interv. (2019) 12:531–40. 10.1016/j.jcin.2018.12.013 30898249

[6] Iwai S Watanabe M Okamura A Kyodo A Nogi K Kamon D . Prognostic impact of calcified plaque morphology after drug eluting stent implantation- an optical coherence tomography study. Circ J. (2021). 85:2019–28. 10.1253/circj.CJ-20-1233 34039823

[7] Aladin AI Al Rifai M Rasool SH Dardari Z Yeboah J Nasir K . Relation of coronary artery calcium and extra-coronary aortic calcium to incident hypertension (from the multi-ethnic study of atherosclerosis). Am J Cardiol. (2018) 121:210–6. 10.1016/j.amjcard.2017.10.018 29174140 PMC5865647

[8] Pawade TA Newby DE Dweck MR. Calcification in aortic stenosis: the skeleton key. J Am Coll Cardiol. (2015) 66:561–77. 10.1016/j.jacc.2015.05.066 26227196

[9] Golub I Lakshmanan S Dahal S Budoff MJ. Utilizing coronary artery calcium to guide statin use. Atherosclerosis. (2021) 326:17–24. 10.1016/j.atherosclerosis.2021.04.011 34000565

[10] Grundy SM Stone NJ Bailey AL Beam C Birtcher KK Blumenthal RS . 2018 AHA/ACC/AACVPR/AAPA/ABC/ACPM/ADA/AGS/APhA/ASPC/NLA/PCNA guideline on the management of blood cholesterol: a report of the American college of cardiology/American heart association task force on clinical practice guidelines. Circulation. (2019) 139:e1082–43. 10.1161/CIR.0000000000000624 30586774 PMC7403606

[11] Nicholson JH. Clinical experiences with anticoagulants; a comparison of coumadin (warfarin) sodium and dicumarol (bishydroxycoumarin). Angiology. (1957) 8:456–65. 10.1177/000331975700800508 13478915

[12] Horton JD Bushwick BM. Warfarin therapy: evolving strategies in anticoagulation. Am Fam Physician. (1999) 59:635–46. 10029789

[13] Ortel TL Neumann I Ageno W Beyth R Clark NP Cuker A . American society of hematology 2020 guidelines for management of venous thromboembolism: treatment of deep vein thrombosis and pulmonary embolism. Blood Advances. (2020) 4:4693–738. 10.1182/bloodadvances.2020001830 33007077 PMC7556153

[14] January CT Wann LS Calkins H Chen LY Cigarroa JE Cleveland JC . 2019 AHA/ACC/HRS focused update of the 2014 AHA/ACC/HRS guideline for the management of patients with atrial fibrillation: a report of the American college of cardiology/American heart association task force on clinical practice guidelines and the heart rhythm society in collaboration with the society of thoracic surgeons. Circulation. (2019) 140:e125–51. 10.1161/CIR.0000000000000665 30686041

[15] Wetmore JB Roetker NS Yan H Reyes JL Herzog CA. Direct-acting oral anticoagulants versus warfarin in medicare patients with chronic kidney disease and atrial fibrillation. Stroke. (2020) 51:2364–73. 10.1161/STROKEAHA.120.028934 32640949

[16] Otto CM Nishimura RA Bonow RO Carabello BA Erwin JP Gentile F . 2020 ACC/AHA guideline for the management of patients with valvular heart disease: a report of the American college of cardiology/American heart association joint committee on clinical practice guidelines. Circulation. (2021) 143:e72–227. 10.1161/CIR.0000000000000923 33332150

[17] Mills C Snider MJ Ortman TC Dush A Hevezi MS Li J . Trends in anticoagulation management services following incorporation of direct oral anticoagulants at a large academic medical center. J Thromb Thrombol. (2021) 51:1050–8. 10.1007/s11239-020-02286-2 33037531 PMC7546384

[18] Willems BA Vermeer C Reutelingsperger CP Schurgers LJ. The realm of vitamin K dependent proteins: shifting from coagulation toward calcification. Mol Nutr Food Res. (2014) 58:1620–35. 10.1002/mnfr.201300743 24668744

[19] Bäck M Aranyi T Cancela ML Carracedo M Conceição N Leftheriotis G . Endogenous calcification inhibitors in the prevention of vascular calcification: a consensus statement from the COST action EuroSoftCalcNet. Front Cardiovasc Med. (2018) 5:196. 10.3389/fcvm.2018.00196 30713844 PMC6345677

[20] Kaesler N Schurgers LJ Floege J. Vitamin K and cardiovascular complications in chronic kidney disease patients. Kidney Int. (2021). 100:1023–36. 10.1016/j.kint.2021.06.037 34310988

[21] Schurgers LJ Teunissen KJ Knapen MH Kwaijtaal M van Diest R Appels A . Novel conformation-specific antibodies against matrix gamma-carboxyglutamic acid (Gla) protein: undercarboxylated matrix Gla protein as marker for vascular calcification. Arterioscl Thromb Vasc Biol. (2005) 25:1629–33. 10.1161/01.ATV.0000173313.46222.43 15961706

[22] van Gorp RH Schurgers LJ. New insights into the pros and cons of the clinical use of vitamin K antagonists (VKAs) versus direct oral anticoagulants (DOACs). Nutrients. (2015) 7:9538–57. 10.3390/nu7115479 26593943 PMC4663607

[23] Sterne JAC Savović J Page MJ Elbers RG Blencowe NS Boutron I . RoB 2: a revised tool for assessing risk of bias in randomised trials. BMJ. (2019) 366:l4898. 10.1136/bmj.l4898 31462531

[24] Wells G SB O'Connell D Peterson J Welch V Losos M Tugwell P. The Newcastle-Ottawa Scale (NOS) for Assessing the Quality of Nonrandomised Studies in Meta-Analyses. (2013). Available online at: https://www.ohri.ca//programs/clinical_epidemiology/oxford.asp (accessed March 29, 2022).

[25] Wilson D. Practical Meta-Analysis Effect Size Calculator [Online calculator] Available online at: https://campbellcollaboration.org/research-resources/effect-size-calculator.html (accessed March 29, 2022).

[26] Suurmond R van Rhee H Hak T. Introduction, comparison, and validation of meta-essentials: a free and simple tool for meta-analysis. Res Synthesis Meth. (2017) 8:537–53. 10.1002/jrsm.1260 28801932 PMC5725669

[27] Andrews J Psaltis PJ Bayturan O Shao M Stegman B Elshazly M . Warfarin use is associated with progressive coronary arterial calcification: insights from serial intravascular ultrasound. JACC Cardiovasc Imag. (2018) 11:1315–23. 10.1016/j.jcmg.2017.04.010 28734922

[28] Chaikriangkrai K Valderrabano M Bala SK Alchalabi S Graviss EA Nabi F . Prevalence and implications of subclinical coronary artery disease in patients with atrial fibrillation. Am J Cardiol. (2015) 116:1219–23. 10.1016/j.amjcard.2015.07.041 26279110

[29] De Vriese AS Caluwe R Pyfferoen L De Bacquer D De Boeck K Delanote J . Multicenter randomized controlled trial of vitamin k antagonist replacement by rivaroxaban with or without vitamin K2 in hemodialysis patients with atrial fibrillation: the valkyrie study. J Am Soc Nephrol. (2020) 31:186–96. 10.1681/ASN.2019060579 31704740 PMC6935010

[30] Hasific S Øvrehus KA Gerke O Hallas J Busk M Lambrechtsen J . Extent of arterial calcification by conventional vitamin K antagonist treatment. PLoS ONE. (2020) 15:e0241450. 10.1371/journal.pone.0241450 33119722 PMC7595268

[31] Koos R Mahnken AH Mühlenbruch G Brandenburg V Pflueger B Wildberger JE . Relation of oral anticoagulation to cardiac valvular and coronary calcium assessed by multislice spiral computed tomography. Am J Cardiol. (2005) 96:747–9. 10.1016/j.amjcard.2005.05.014 16169351

[32] Lee J Nakanishi R Li D Shaikh K Shekar C Osawa K . Randomized trial of rivaroxaban versus warfarin in the evaluation of progression of coronary atherosclerosis. Am Heart J. (2018) 206:127–30. 10.1016/j.ahj.2018.08.007 30227941

[33] Palaniswamy C Aronow WS Sekhri A Adapa S Ahn C Singh T . Warfarin use and prevalence of coronary artery calcification assessed by multislice computed tomography. Am J Ther. (2014) 21:148–51. 10.1097/MJT.0b013e318249a1c6 22820716

[34] Plank F Beyer C Friedrich G Stühlinger M Hintringer F Dichtl W . Influence of vitamin K antagonists and direct oral anticoagulation on coronary artery disease: a CTA analysis. Int J Cardiol. (2018) 260:11–5. 10.1016/j.ijcard.2018.03.019 29530620

[35] Schurgers LJ Joosen IA Laufer EM Chatrou ML Herfs M Winkens MH . Vitamin K-antagonists accelerate atherosclerotic calcification and induce a vulnerable plaque phenotype. PLoS ONE. (2012) 7:e43229. 10.1371/journal.pone.0043229 22952653 PMC3430691

[36] Ünlü S Sahinarslan A Kiliç HK Gökalp G Sezenöz B Erbaş G . Long-term vitamin-K antagonist use and coronary artery calcification. Herz. (2020) 45:580–5. 10.1007/s00059-018-4760-9 30276478

[37] Villines TC O'Malley PG Feuerstein IM Thomas S Taylor AJ. Does prolonged warfarin exposure potentiate coronary calcification in humans? Results of the warfarin and coronary calcification study. Calcif Tissue Int. (2009) 85:494–500. 10.1007/s00223-009-9300-4 19847375

[38] Weijs B Blaauw Y Rennenberg RJ Schurgers LJ Timmermans CC Pison L . Patients using vitamin K antagonists show increased levels of coronary calcification: an observational study in low-risk atrial fibrillation patients. Eur Heart J. (2011) 32:2555–62. 10.1093/eurheartj/ehr226 21775389

[39] Win TT Nakanishi R Osawa K Li D Susaria SS Jayawardena E . Apixaban versus warfarin in evaluation of progression of atherosclerotic and calcified plaques (prospective randomized trial). Am Heart J. (2019) 212:129–33. 10.1016/j.ahj.2019.02.014 31002997

[40] Alappan HR Kaur G Manzoor S Navarrete J O'Neill WC. Warfarin accelerates medial arterial calcification in humans. Arterioscl Thromb Vasc Biol. (2020) 40:1413–9. 10.1161/ATVBAHA.119.313879 32078340

[41] Eren Sadioglu R Ustuner E Ergun I Ecder ST Nergizoglu G Keven K. Warfarin is associated with the risk of vascular calcification in abdominal aorta in hemodialysis patients: a multicenter case-control study. Turk J Med Sci. (2021). 51:2607–15. 10.3906/sag-2104-221 34289653 PMC8742472

[42] Fusaro M Tripepi G Noale M Plebani M Zaninotto M Piccoli A . Prevalence of vertebral fractures, vascular calcifications, and mortality in warfarin treated hemodialysis patients. Curr Vasc Pharmacol. (2015) 13:248–58. 10.2174/15701611113119990146 23927679

[43] Fusaro M Gallieni M Rebora P Rizzo MA Luise MC Riva H . Atrial fibrillation and low vitamin D levels are associated with severe vascular calcifications in hemodialysis patients. J Nephrol. (2016) 29:419–26. 10.1007/s40620-015-0236-7 26493621

[44] Han KH Hennigar RA O'Neill WC. The association of bone and osteoclasts with vascular calcification. Vasc Med. (2015) 20:527–33. 10.1177/1358863X15597076 26324151

[45] Han KH O'Neill WC. Increased peripheral arterial calcification in patients receiving warfarin. J Am Heart Assoc. (2016) 5:e002665. 10.1161/JAHA.115.002665 26811161 PMC4859382

[46] Jean G Bresson E Terrat JC Vanel T Hurot JM Lorriaux C . Peripheral vascular calcification in long-haemodialysis patients: associated factors and survival consequences. Nephrol Dial Transplant. (2009) 24:948–55. 10.1093/ndt/gfn571 18852190

[47] Jean G Chazot C Bresson E Zaoui E Cavalier E. High serum sclerostin levels are associated with a better outcome in haemodialysis patients. Nephron. (2016) 132:181–90. 10.1159/000443845 26890570

[48] Nuotio K Koskinen SM Mäkitie L Tuimala J Ijäs P Heikkilä HM . Warfarin treatment is associated to increased internal carotid artery calcification. Front Neurol. (2021) 12:696244. 10.3389/fneur.2021.696244 34322086 PMC8311519

[49] Peeters F Dudink E Kimenai DM Weijs B Altintas S Heckman LIB . Vitamin K antagonists, non-vitamin K antagonist oral anticoagulants, and vascular calcification in patients with atrial fibrillation. TH Open. (2018) 2:e391–e8. 10.1055/s-0038-1675578 31249966 PMC6524908

[50] Peeters MTJ Houben R Postma AA van Oostenbrugge RJ Schurgers LJ Staals J. Vitamin K antagonist use and risk for intracranial carotid artery calcification in patients with intracerebral hemorrhage. Front Neurol. (2019) 10:1278. 10.3389/fneur.2019.01278 31920910 PMC6933022

[51] Rennenberg RJ van Varik BJ Schurgers LJ Hamulyak K Ten Cate H Leiner T . Chronic coumarin treatment is associated with increased extracoronary arterial calcification in humans. Blood. (2010) 115:5121–3. 10.1182/blood-2010-01-264598 20354170

[52] Tantisattamo E Han KH O'Neill WC. Increased vascular calcification in patients receiving warfarin. Arterioscl Thromb Vasc Biol. (2015) 35:237–42. 10.1161/ATVBAHA.114.304392 25324574

[53] Van Berkel B Van Ongeval C Van Craenenbroeck AH Pottel H De Vusser K Evenepoel P. Prevalence, progression and implications of breast artery calcification in patients with chronic kidney disease. Clin Kidney J. (2022) 15:295–302. 10.1093/ckj/sfab178 35145644 PMC8825218

[54] Wei N Lu L Zhang H Gao M Ghosh S Liu Z . Warfarin accelerates aortic calcification by upregulating senescence-associated secretory phenotype maker expression. Oxid Med Cell Longev. (2020) 2020:2043762. 10.1155/2020/2043762 33149806 PMC7603623

[55] Di Lullo L Tripepi G Ronco C D'Arrigo G Barbera V Russo D . Cardiac valve calcification and use of anticoagulants: preliminary observation of a potentially modifiable risk factor. Int J Cardiol. (2019) 278:243–9. 10.1016/j.ijcard.2018.11.119 30538058

[56] Ing SW Mohler Iii ER Putt ME Torigian D Leonard MB. Correlates of valvular ossification in patients with aortic valve stenosis. Clin Transl Sci. (2009) 2:431–5. 10.1111/j.1752-8062.2009.00168.x 20443935 PMC5350723

[57] Koos R Krueger T Westenfeld R Kühl HP Brandenburg V Mahnken AH . Relation of circulating matrix gla-protein and anticoagulation status in patients with aortic valve calcification. Thromb Haemost. (2009) 101:706–13. 10.1160/TH08-09-0611 19350115

[58] Lerner RG Aronow WS Sekhri A Palaniswamy C Ahn C Singh T . Warfarin use and the risk of valvular calcification. J Thromb Haemost. (2009) 7:2023–7. 10.1111/j.1538-7836.2009.03630.x 19793187

[59] Sonderskov PS Lindholt JS Hallas J Gerke O Hasific S Lambrechtsen J . Association of aortic valve calcification and vitamin K antagonist treatment. Eur Heart J Cardiovasc Imag. (2020) 21:718–24. 10.1093/ehjci/jeaa065 32361722

[60] Tastet L Pibarot P Shen M Clisson M Côté N Salaun E . Oral anticoagulation therapy and progression of calcific aortic valve stenosis. J Am Coll Cardiol. (2019) 73:1869–71. 10.1016/j.jacc.2019.01.043 30975306

[61] Yamamoto K Koretsune Y Akasaka T Kisanuki A Ohte N Takenaka T . Effects of vitamin K antagonist on aortic valve degeneration in non-valvular atrial fibrillation patients: prospective 4-year observational study. Thromb Res. (2017) 160:69–75. 10.1016/j.thromres.2017.10.027 29121522

[62] Kauppila L Polak J Cupples L Hannan M Kiel D Wilson P. New indices to classify location, severity and progression of calcific lesions in the abdominal aorta: a 25- year follow-up study. Atherosclerosis. (1997) 132:245–50. 10.1016/S0021-9150(97)00106-8 9242971

[63] Witteman JC Grobbee DE Valkenburg HA van Hemert AM Stijnen T Burger H . J-shaped relation between change in diastolic blood pressure and progression of aortic atherosclerosis. Lancet. (1994) 343:504–7. 10.1016/S0140-6736(94)91459-1 7906758

[64] Levy RJ Schoen FJ Levy JT Nelson AC Howard SL Oshry LJ. Biologic determinants of dystrophic calcification and osteocalcin deposition in glutaraldehyde-preserved porcine aortic valve leaflets implanted subcutaneously in rats. Am J Pathol. (1983) 113:143–55. 6605687 PMC1916380

[65] Taybi H Capitanio MA. Tracheobronchial calcification: an observation in three children after mitral valve replacement and warfarin sodium therapy. Radiology. (1990) 176:728–30. 10.1148/radiology.176.3.2389031 2389031

[66] Yoshida S Torikai K. The effects of warfarin on calcinosis in a patient with systemic sclerosis. J Rheumatol. (1993) 20:1233–5. 8371227

[67] Jurgens PT Carr JJ Terry JG Rana JS Jacobs DR Duprez DA. Association of abdominal aorta calcium and coronary artery calcium with incident cardiovascular and coronary heart disease events in black and white middle‐ aged people: the coronary artery risk development in young adults study. J Am Heart Assoc. (2021) 10:e023037. 10.1161/JAHA.121.023037 34873926 PMC9075251

[68] Takayama Y Yasuda Y Suzuki S Shibata Y Tatami Y Shibata K . Relationship between abdominal aortic and coronary artery calcification as detected by computed tomography in chronic kidney disease patients. Heart Vessels. (2016) 31:1030–7. 10.1007/s00380-015-0712-y 26164596

[69] Minssen L Dao TH Quang AV Martin L Andureau E Luciani A . Breast arterial calcifications on mammography: a new marker of cardiovascular risk in asymptomatic middle age women? Eur Radiol. (2022) 32:4889–97. 10.1007/s00330-022-08571-3 35147775

[70] Nasir K Katz R Al-Mallah M Takasu J Shavelle DM Carr JJ . Relationship of aortic valve calcification with coronary artery calcium severity: the multi-ethnic study of atherosclerosis (MESA). J Cardiovasc Comp Tomog. (2010) 4:41–6. 10.1016/j.jcct.2009.12.002 20159627

[71] Tintut Y Honda HM Demer LL. Biomolecules orchestrating cardiovascular calcification. Biomolecules. (2021) 11:1482. 10.3390/biom11101482 34680115 PMC8533507

[72] Aikawa E Blaser MC Jeffrey M. Hoeg award lecture: calcifying extracellular vesicles as building blocks of microcalcifications in cardiovascular disorders. Arterioscl Thromb Vasc Biol. (2021) 41:117–27. 10.1161/ATVBAHA.120.314704 33115271 PMC7832175

[73] Kim KM. Calcification of matrix vesicles in human aortic valve and aortic media. Fed Proc. (1976) 35:156–62. 1248649

[74] Bertazzo S Gentleman E Cloyd KL Chester AH Yacoub MH Stevens MM. Nano-analytical electron microscopy reveals fundamental insights into human cardiovascular tissue calcification. Nat Mater. (2013) 12:576–83. 10.1038/nmat3627 23603848 PMC5833942

[75] Abdelbaky A Corsini E Figueroa AL Fontanez S Subramanian S Ferencik M . Focal arterial inflammation precedes subsequent calcification in the same location: a longitudinal FDG-PET/CT study. Circ Cardiovasc Imag. (2013) 6:747–54. 10.1161/CIRCIMAGING.113.000382 23833282

[76] Chowdhury MM Tarkin JM Albaghdadi MS Evans NR Le EPV Berrett TB . Vascular positron emission tomography and restenosis in symptomatic peripheral arterial disease: a prospective clinical study. JACC Cardiovasc Imag. (2020) 13:1008–17. 10.1016/j.jcmg.2019.03.031 31202739 PMC7136751

[77] Dweck MR Jones C Joshi NV Fletcher AM Richardson H White A . Assessment of valvular calcification and inflammation by positron emission tomography in patients with aortic stenosis. Circulation. (2012) 125:76–86. 10.1161/CIRCULATIONAHA.111.051052 22090163

[78] Bjørklund G Svanberg E Dadar M Card DJ Chirumbolo S Harrington DJ . The role of matrix gla protein (MGP) in vascular calcification. Curr Med Chem. (2020) 27:1647–60. 10.2174/0929867325666180716104159 30009696

[79] Vasan RS Short MI Niiranen TJ Xanthakis V DeCarli C Cheng S . Interrelations between arterial stiffness, target organ damage, and cardiovascular disease outcomes. J Am Heart Assoc. (2019) 8:e012141. 10.1161/JAHA.119.012141 31303106 PMC6662123

[80] Lee GP Kim HL. Incremental value of the measures of arterial stiffness in cardiovascular risk assessment. Rev Cardiovasc Med. (2022) 23:6. 10.31083/j.rcm2301006 35092198

[81] Vasan RS Pan S Xanthakis V Beiser A Larson MG Seshadri S . Arterial stiffness and long-term risk of health outcomes: the framingham heart study. Hypertension. (2022) 79:1045–56. 10.1161/HYPERTENSIONAHA.121.18776 35168368 PMC9009137

[82] Maroules CD Khera A Ayers C Goel A Peshock RM Abbara S . Cardiovascular outcome associations among cardiovascular magnetic resonance measures of arterial stiffness: the dallas heart study. J Cardiovasc Magn Reson. (2014) 16:33. 10.1186/1532-429X-16-33 24886531 PMC4031496

[83] Virmani R Kolodgie FD Burke AP Farb A Schwartz SM. Lessons from sudden coronary death: a comprehensive morphological classification scheme for atherosclerotic lesions. Arterioscl Thromb Vasc Biol. (2000) 20:1262–75. 10.1161/01.ATV.20.5.1262 10807742

[84] Høilund-Carlsen PF Sturek M Alavi A Gerke O. Atherosclerosis imaging with (18)F-sodium fluoride PET: state-of-the-art review. Eur J Nucl Med Mol Imag. (2020) 47:1538–51. 10.1007/s00259-019-04603-1 31773235 PMC7188711

[85] Nishimura S Izumi C Nishiga M Amano M Imamura S Onishi N . Predictors of rapid progression and clinical outcome of asymptomatic severe aortic stenosis. Circ J. (2016) 80:1863–9. 10.1253/circj.CJ-16-0333 27334030

[86] Rosenhek R Binder T Porenta G Lang I Christ G Schemper M . Predictors of outcome in severe, asymptomatic aortic stenosis. N Engl J Med. (2000) 343:611–7. 10.1056/NEJM200008313430903 10965007

[87] Poterucha TJ Goldhaber SZ. Warfarin and vascular calcification. Am J Med. (2016) 129:635.e1–4. 10.1016/j.amjmed.2015.11.032 26714212

