# Supplementary material for: Vitamin K antagonists and cardiovascular calcification: A systematic review and meta-analysis
Source: Front Cardiovasc Med. 2022 Aug 19;9:938567. doi: 10.3389/fcvm.2022.938567 (PMC9437425; doi:10.3389/fcvm.2022.938567)
Supplement: Supplementary file 1 [file Data_Sheet_1.pdf]

Supplementary Material

1 Supplementary Tables

1.1 Supplementary Table 1 Pubmed search conducted on March 29, 2022

Search	Terms	Results
Treatment or exposure	"vitamin k antagonist"[All Fields] OR "VKA"[All Fields] OR "warfarin"[All Fields] OR "coumarin"[All Fields] OR "acenocoumarol"[All Fields] OR "phenprocoumon"[All Fields] OR "fluindione"[All Fields]	50,060
Patients	"atrial fibrillation"[tw] OR "valvular heart disease*"[tw] OR "artificial heart valve*"[tw] OR "pulmonary embolism"[tw] OR "deep venous thrombosis"[tw] OR "antiphospholipid syndrome"[tw] OR "myocardial infarction*"[tw] OR "ischemic stroke*"[tw] OR "coronary artery disease*"[tw] OR "peripheral arterial disease"[tw] OR "PAD" OR "venous thromboembolism"[tw] OR "prosthetic heart valve*"[tw] OR "rheumatic valve disease*"[tw] OR "cryptogenic stroke"[tw] OR "patent foramen ovale"[tw] OR patient*[tw] OR human*[tw] OR subject*[tw] OR participant*[tw]	22,398,219
Outcomes	"vascular calcification"[MeSH] OR "coronary artery calc*"[tw] OR "aortic calc*"[tw] OR "aortic valve calcification*"[tw] OR "calcific aortic valve stenosis"[tw] OR "thoracic aortic calc*"[tw] OR "abdominal vascular calc*"[tw] OR "vascular calc*"[tw] OR "breast arterial calc*"[tw] OR "peripheral arterial disease"[tw] OR "peripheral artery calc*"[tw] OR "renal artery calc*"[tw] OR "intracranial artery calc*"[tw] OR "monckeberg medial calcific sclerosis"[tw]	33,013
	1 AND 2 AND 3	330

1.2 **Supplementary Table 2.** Assessment of risk of bias in the included randomized trials

				Randomization	Deviations from interventions	Missing outcome data	Outcome measurement	Selective reporting	
**Analysis**	**Study**	**VKA**	**No VKA**	**D1**	**D2**	**D3**	**D4**	**D5**	**Overall**
Intention-to-treat	De Vriese et al. 2020	VKA	Rivaroxaban						
Per-protocol	Lee et al. 2018	Warfarin	Rivaroxaban						
Per-protocol	Win et al. 2019	Warfarin	Apixaban						

Risk of bias scale

Low risk

Some concerns

High risk

Revised Cochrane risk of bias tool for randomized trials (RoB 2). Version - August 22, 2019 https://sites.google.com/site/riskofbiastool/welcome/rob-2-0-tool/current-version-of-rob-2(Excel Macro Form, version 9 - downloaded February 9, 2022)

1.3 **Supplementary Table 3.** Modified Newcastle – Ottawa quality assessment scale

	Selection				Comparability		Outcome			
	Representativeness of exposed subjects	Selection of non-exposed subjects	Ascertainment of exposure	Outcome was not present at baseline	Controlled for age	Controlled for another calcification factor	Assessment of outcomes	Was follow-up long enough	Enrolled subjects' follow-up	Total score
Coronary										
Andrews et al. 2018	*	*	*	*	*	*	*	*	*	9
Chaikriangkrai et al. 2015	*	*	*	*			*			5
Hasific et al. 2020	*	*	*	*	*	*	*	*	*	9
Koos et al. 2005 (a)		*	*				*	*		4
Palaniswamy et al. 2014	*	*	*				*			4
Plank et al. 2018	*	*	*	*	*	*	*	*	*	9
Schurgers et al. 2012 (a)	*	*	*		*	*	*		*	7
Schurgers et al. 2012 (b)	*	*	*		*	*	*	*	*	8
Schurgers et al. 2012 (c)	*	*	*		*	*	*	*	*	8
Unlu et al. 2020	*	*	*		*	*		*	*	7
Villines et al. 2009	*	*	*	*			*	*	*	7
Weijs et al. 2011	*	*	*	*	*	*	*	*	*	9
Extra-coronary										
Alappan et al. 2020 (a)	*	*	*				*	*	*	6

	Selection				Comparability		Outcome			
	Representativeness of exposed subjects	Selection of non-exposed subjects	Ascertainment of exposure	Outcome was not present at baseline	Controlled for age	Controlled for another calcification factor	Assessment of outcomes	Was follow-up long enough	Enrolled subjects' follow-up	Total score
Alappan et al. 2020 (b)	*	*	*				*	*	*	6
Alappan et al. 2020 (c)	*	*	*				*	*	*	6
Eren-Sadioglu et al. 2021	*	*	*		*	*	*	*	*	8
Fusaro et al. 2015	*	*	*		*	*	*	*	*	8
Fusaro et al. 2016	*	*	*		*	*	*			6
Han et al. 2015	*	*	*				*			4
Han et al. 2016	*	*	*		*	*	*	*	*	8
Jean et al. 2009	*	*	*		*	*				5
Jean et al. 2016	*	*	*							3
Nuotio et al. 2021	*	*	*		*	*	*	*	*	8
Peeters et al. 2018	*	*	*	*	*	*	*	*	*	9
Peeters et al. 2019	*	*	*		*	*	*	*	*	8
Rennenberg et al. 2010	*	*	*	*	*	*	*	*	*	9
Tantisattamo et al. 2015	*	*	*	*	*	*	*	*	*	9
Van Berkel et al. 2022	*	*	*				*			4

	Selection				Comparability		Outcome			
	Representativeness of exposed subjects	Selection of non-exposed subjects	Ascertainment of exposure	Outcome was not present at baseline	Controlled for age	Controlled for another calcification factor	Assessment of outcomes	Was follow-up long enough	Enrolled subjects' follow-up	Total score
Wei et al. 2020	*	*	*		*	*	*		*	7
Aortic valve										
Di Lullo et al. 2019	*	*	*	*		*		*	*	7
Ing et al. 2009	*	*	*		*	*	*			6
Koos et al. 2005 (b)		*	*				*	*	*	5
Koos et al. 2009	*	*	*		*	*		*	*	7
Lerner et al. 2009		*	*		*	*	*			6
Peeters et al. 2018	*	*	*		*	*				5
Sonderskov et al. 2020	*	*	*		*	*	*	*	*	8
Tastet et al. 2019	*	*	*	*	*	*	*	*	*	9
Yamamoto et al. 2017	*	*	*	*			*	*		6

SELECTION – maximum 4 stars (****)
Representativeness of the exposed subjects
a. Exposed subjects are representative of exposed individuals in the community *
b. Exposed subjects are drawn from a unique population (e.g., medical workers, self-selected volunteers)
c. No description

Selection of the non-exposed cohort/subjects
a. Drawn from the same community as the exposed *
b. Drawn from a different source than the exposed
c. No description

Ascertainment of exposure
a. Objective medical record *

b. Self-report
c. No description

Demonstration that outcome of interest was not present at baseline (maximum 1 star)

a. Demonstrated *
b. Stated no known history of CAD or vascular disease *
c. Not documented
d. A cross-sectional study – no baseline

COMPARABILITY – maximum 2 stars (**)

Comparability of exposed and non-exposed subjects

a. Controlled for age *
b. Controlled for at least one other confounding factor associated with calcification (male sex, smoking, diabetes, reduced renal function, hypertension) *

OUTCOME – maximum 3 stars (***)

Unbiased assessment of outcomes (maximum 1 star)

a. Blind assessment *
b. Secure record *
c. Self-reported
d. No description.

Was the follow up long enough for outcomes to occur

a. Median follow up is greater or equal to 6 months *
b. The median follow up was less than 6 months
c. Cross-sectional study – no follow-up

Adequacy of follow up of the enrolled subjects

a. Follow up rate is greater or equal to 90% *
b. Follow up rate is less than 90%
c. Cross-sectional study – no follow-up

1.4 Supplementary Table 4. Sensitivity analysis by the exclusion of individual studies

Excluded study	Weight	OR [95% CI]	p-value	I^2
Coronary				
none	100%	1.21 [1.08, 1.36]	0.001	68%
Andrews et al. 2018	18.7%	1.25 [1.08, 1.44]	0.003	67%
Chaikriangkrai et al. 2015	6.1%	1.20 [1.07, 1.35]	0.003	68%
De Vriese et al. 2020 (a)	3.2%	1.20 [1.07, 1.35]	0.003	68%
Hasific et al. 2020	21.5%	1.27 [1.11, 1.46]	0.0006	50%
Koos et al. 2005 (a)	1.6%	1.18 [1.06, 1.30]	0.002	61%
Lee et al. 2018	12.5%	1.20 [1.06, 1.36]	0.0008	67%
Palaniswamy et al. 2014	1.9%	1.22 [1.09, 1.38]	0.0008	70%
Plank et al. 2018	4.3%	1.22 [1.08, 1.37]	0.001	70%
Schurgers et al. 2012 (a)	2.1%	1.23 [1.10, 1.38]	0.0004	68%
Schurgers et al. 2012 (b)	2.1%	1.22 [1.08, 1.37]	0.001	70%
Schurgers et al. 2012 (c)	2.2%	1.20 [1.07, 1.35]	0.002	69%
Unlu et al. 2020	2.4%	1.18 [1.06, 1.32]	0.003	65%
Villines et al. 2009	1.4%	1.20 [1.07, 1.34]	0.002	68%
Weijs et al. 2011	1.2%	1.20 [1.07, 1.34]	0.002	67%
Win et al. 2019	18.9%	1.25 [1.08, 1.45]	0.003	67%
Extra-coronary				
none	100%	1.86 [1.43, 2.42]	< 0.00001	78%
Alappan et al. 2020 (a)	5.2%	1.77 [1.37, 2.29]	< 0.0001	76%
Alappan et al. 2020 (b)	5.4%	1.85 [1.41, 2.41]	< 0.00001	79%
Alappan et al. 2020 (c)	3.4%	1.82 [1.40, 2.37]	< 0.00001	79%
De Vriese et al. 2020(b)	2.0%	1.88 [1.44, 2.46]	< 0.00001	80%
Fusaro et al. 2015	3.5%	1.82 [1.40, 2.38]	< 0.00001	78%

Excluded study	Weight	OR [95% CI]	p-value	I^2
Fusaro et al. 2016	5.4%	1.95 [1.49, 2.55]	< 0.00001	78%
Han et al. 2015	3.9%	1.88 [1.44, 2.46]	< 0.00001	79%
Han et al. 2016	3.2%	1.90 [1.43, 2.52]	< 0.00001	79%
Jean et al. 2009	8.2%	1.88 [1.44, 2.46]	< 0.00001	79%
Jean et al. 2016	4.0%	1.79 [1.38, 2.32]	< 0.0001	77%
Nuotio et al 2021	4.5%	1.81 [1.39, 2.35]	< 0.0001	81%
Peeters et al. 2018	6.8%	1.84 [1.40, 2.40]	< 0.00001	78%
Peeters et al. 2019	5.9%	1.89 [1.42, 2.51]	< 0.0001	78%
Rennenberg et al. 2010	8.4%	1.89 [1.42, 2.51]	< 0.00001	78%
Sadioglu et al. 2021	8.5%	1.81 [1.39, 2.36]	< 0.00001	78%
Tantisattamo et al. 2015	9.8%	1.95 [1.54, 2.46]	< 0.00001	50%
Van Berkel et al. 2022	4.6%	1.78 [1.38, 2.31]	< 0.0001	77%
Wei et al. 2020	7.3%	1.99 [1.50, 2.63]	< 0.00001	79%
Aortic valve				
none	100%	3.07 [1.90, 4.96]	< 0.00001	90%
Di Lullo et al. 2019	9.0%	2.44 [1.58, 3.76]	< 0.0001	87%
Ing et al. 2009	7.2%	2.91 [1.78, 4.74]	< 0.0001	91%
Koos et al. 2005(b)	10.1%	2.75 [1.71, 4.43]	< 0.0001	89%
Koos et al. 2009	11.5%	3.17 [1.89, 5.31]	< 0.0001	91%
Lerner et al. 2009	15.3%	3.83 [1.78, 8.24]	0.0006	91%
Peeters et al. 2018	12.2%	3.30 [1.95, 5.59]	< 0.00001	91%
Sonderskov et al. 2020	15.9%	3.90 [2.11, 7.21]	< 0.0001	82%
Tastet et al. 2019	8.5%	2.74 [1.70, 4.42]	< 0.0001	90%
Yamamoto et al. 2017	10.3%	3.08 [1.85, 5.11]	< 0.0001	91%
